# Context-Aware Mobile Collaborative Systems: Conceptual Modeling and Case Study

**DOI:** 10.3390/s121013491

**Published:** 2012-10-09

**Authors:** Edgard Benítez-Guerrero, Carmen Mezura-Godoy, Luis G. Montané-Jiménez

**Affiliations:** Facultad de Estadística e Informática, Universidad Veracruzana, Xalapa 91020, Mexico; E-Mails: cmezura@uv.mx (C.M.-G.); lmontane@uv.mx (L.G.M.-J.)

**Keywords:** mobile collaborative system, context-awareness, activity model

## Abstract

A Mobile Collaborative System (MCOS) enable the cooperation of the members of a team to achieve a common goal by using a combination of mobile and fixed technologies. MCOS can be enhanced if the *context* of the group of users is considered in the execution of activities. This paper proposes a novel model for Context-Aware Mobile COllaborative Systems (CAMCOS) and a functional architecture based on that model. In order to validate both the model and the architecture, a prototype system in the tourism domain was implemented and evaluated.

## 1. Introduction

A Mobile Collaborative System (MCOS) enable the members of a team to communicate, collaborate and coordinate their activities in order to achieve a common goal, in a loosely-coupled manner, by using a combination of mobile and fixed technologies [1]. For instance, a mobile tourist guide system can support groups of nomad tourists to achieve a collaborative learning task, such as learning the history of a city downtown, by enabling tourists to interchange relevant knowledge and experiences via messaging, and to coordinate their activities through their participation in virtual meetings. Traditional MCOS provide their users with information and services in predetermined ways: the same data items are presented in the same way to all the users, and the same services are invoked in the same sequence. Although this behaviour is enough for some applications, a MCOS should satisfy more effectively the information/services needs of its users. Imagine the following example scenario.

Carmen and Luis are two young tourists that have just arrived to Xalapa City, Mexico, well known by its parks and gardens, and its cultural heritage. At the tourist office, they are proposed to participate in a team competition to win a prize. The objective of the competition is to answer a set of questions relating points of interest (POIs) located in the city downtown before the other teams participating in the contest. The participants are not alone: each of them is provided with a mobile device containing an application called Bon Voyage (BV for short) where the tourist can see and answer the questionnaire. This application provides also functionalities such as mapping tools to locate POIs and the other tourists participating, virtual note sharing, clues to let the user know the POIs that are involved in a question, and notifications about the activities performed by other tourists; e.g., sharing a note, viewing a map, answering a question. It also provides a Virtual Note Viewer (VNV), for reading virtual notes attached to POIs, which is smart enough to adapt itself according to the current physical activity of the user; e.g., if the user is facing a POI and points the mobile to it, VNV uses Augmented Reality (AR) to present the text note attached to the POI, instead of the default normal window showing the text. Carmen and Luis consider that this is a fun way to learn about the history of the city and decide to enter the competition. As they need to minimize time, they decide to visit POIs in parallel, read the information attached to each POI, and share this information with each other in order to answer the questions. This strategy worked well for them and finally won the prize. Later, they returned to the places they liked the most.

In this scenario, the provided mobile collaborative application is not a regular one: it is able to detect user and environmental states (*i.e.*, the context) and use this data to provide adequate and relevant data and/or services; *i.e.*, it is *context-aware* [2]. Particularly, context is used to provide domain-specific information to a user, such as the informative virtual notes attached to the POIs, or group-dependent information, such as the current location of other tourists and the activities they are doing. Context is also used to adapt the user interface of the virtual note viewer as required. Context-Aware Mobile COllaborative Systems (CAMCOS) have been studied by more than one decade but now, with the advances on mobile and ubiquitous computing, the subject has regained attention.

**Figure 1.**
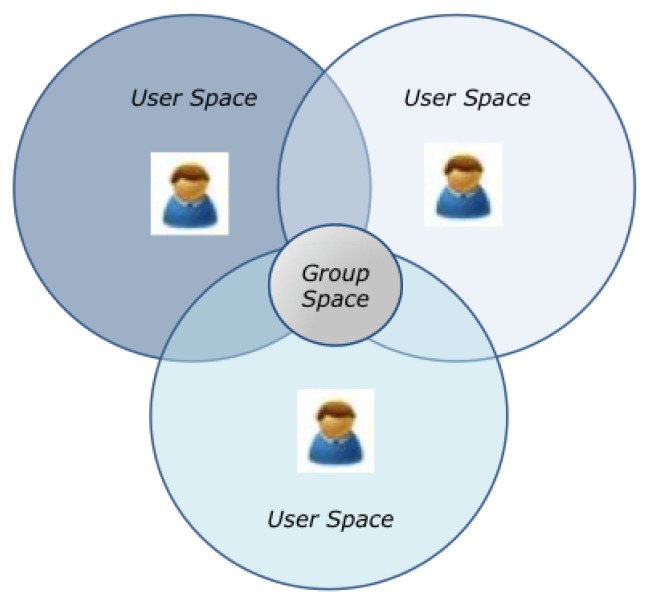
User and group spaces.

CAMCOS are important because it is expected that they will help to increase the knowledge and productivity of their users. However, there are several challenges that must be faced in order to have this kind of systems: context modeling, acquisition and usage, group and activity modeling, and system design, implementation and testing. This paper proposes a model for CAMCOS, based on the concepts of User Space and Group Space (see Figure 1), which characterizes users, their individual and group activities, the execution context of those activities, and how this context can affect them. It also proposes a functional architecture for CAMCOS based on the model. In order to validate both the model and the functional architecture, a prototype of the Bon Voyage system was implemented and evaluated. Note that, although our case study is about collaborative learning in the Tourism domain, the proposed model and functional architecture are domain-independent and can be applied to other application areas, such as collaborative decision making in a business setting. This paper is organized as follows. Section 2 introduces our model for CAMCOS, while Section 3 explains our proposal for a functional architecture. Section 4 introduces BV, and Section 5 reports an exploratory study we conducted to measure potential user satisfaction and system performance. Section 6 discusses related work. Finally, Section 7 concludes this paper.

## 2. A Model for CAMCOS

This Section proposes a model for CAMCOS, which extends our previous work [3], that is based on the notions of *User Space* and *Group Space*, and in the elements summarized in Table 1. The following paragraphs explain these concepts in detail.

### 2.1. User Spaces

**Figure 2.**
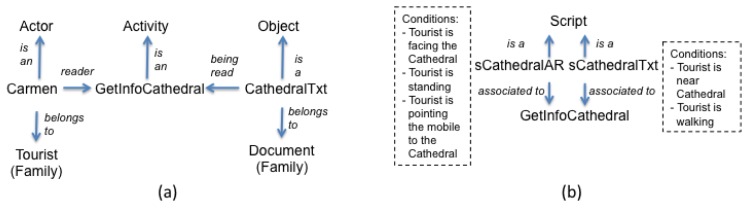
A User Space and its elements.

Conceptually, it is possible to associate a *space* to each user (see Figure 1). It is within this space where the user executes his/her activities and, it is this space which determines the execution context of the activities. Throughout an *activity*, users (also called *actors*) manipulate and produce *objects*. A set of actors or objects having the same features is called a *family*. Consider for instance that, to answer the question Q1 “Name of the personality who was buried in the Old Cemetery on 1938, but now is buried in the Metropolitan Cathedral”, Carmen needs information about the city's cathedral, so she goes there while Luis goes to the Cemetery. When she arrives, she uses BV's viewer to read the note “St Guizar y Valencia was buried in Cathedral on October 24, 1950”. In this case, the actor “Carmen” of the family “Tourist” executes the activity “GetInfoCathedral”, in which the object “CathedralTxt” of the family “Document” containing information about the cathedral is manipulated. During an activity, actors and objects play *roles*. For instance, during the activity “GetInfoCathedral”, the actor “Carmen” plays the role “reader”, and the object “CathedralTxt” plays the role “Being Read”. Figure 2(a) represents these concepts.

Associated to each activity, there is one or more *scripts*. A script describes the way an activity must be executed. For the activity “GetInfoCathedral” it is possible to associate two scripts (see Figure 2(b)): one for displaying notes in the VNV using its text-based version (“sCathedralTxt”) and other for the same purpose but using its AR-based version (“sCathedralAR”). A script can have pre- and post- conditions that need to be satisfied to execute it. For instance, the script “sCathedralAR” can include as pre-conditions that (1) the tourist must be facing the Cathedral; (2) the tourist must stand; and (3) the tourist must be pointing the mobile device to the Cathedral. As the pre-conditions are met when Carmen arrives to the Cathedral, she can see the note in an AR view.

An activity model is a general description of an activity; *i.e.*, an activity is a particular instance of an activity model. It describes the family of actors that can participate in the activity, how an activity can be carried out, the family of objects that can be manipulated or produced, and which roles will play actors and objects in the activity. Table 2 shows the activity model for all the GetInfoCathedral activities.

As previously said, the space of a user determines the execution context of his/her activities. As usual, contextual information can be categorized as human-centered factors (e.g., personal habits, social awareness) and environmental factors (e.g., location, light conditions), and the variables of the context to be considered are application-dependent. The important aspect to be considered here is that a particular context can determine the script to be run for an activity. For the activity “GetInfoCathedral”, the script to display notes in an AR view is appropriate when the user is in front of the cathedral and is pointing his/her mobile device to it, but in any other case a regular window displaying text notes is enough. Note also that, no matter which script is selected to run the activity, the current user's location can be used to automatically choose the information to be shown, in this case data about the cathedral.

### 2.2. Group Spaces

**Figure 3.**
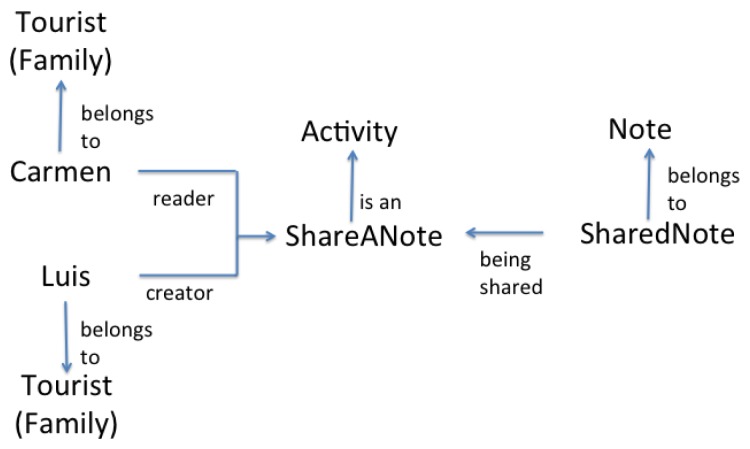
A Group Space and its elements.

Actors can also participate in group activities in order to achieve common goals. A group activity is executed in a *Group Space*, which is the intersection of the user spaces of the actors participating in the activity (see Figure 1). Imagine that Luis arrives to the Cemetery and read a virtual note that says “Distinguished personalities are buried here, such as Enrique C. Rebsamen. The first tomb of St Guizar y Valencia is also here”. At that moment, Luis writes a note to share this information with Carmen. The activity “ShareANote” (see Figure 3) has “Carmen” and “Luis” as actors, both belonging to the family “Tourist”. The object in the activity is a “sharedNote” from the family “Note”, which contains a message and has a temporal timestamp associated (e.g., “St. Guizar y Valencia is buried here!” at 11:30 AM). When the activity starts, Luis plays the role “Creator”, and Carmen plays the role “Reader”, while the shared note plays the role “Being Shared”. Associated to the activity, one can find the script “scriptShareANote”, which indicates that the note will be broadcast to the other members of a group. The script has a pre-condition which indicates that the note will be sent only if the current time is earlier than 9:00 PM. As this pre-condition is satisfied, Carmen receives the note. After reading Luis' notes, Carmen learns that St. Guizar y Valencia was buried in the Old Cemetery and, as she already knows that his remains lies now at the Cathedral, she can answer correctly the question Q1.

The activities in a group space are also influenced by the current context, particularly by human factors as social awareness. When an actor *t*_1_ executes an activity *a* and this fact is known by actor *t*_2_, it is possible that *t*_2_ will stop doing his/her current activity and will start executing *a* or another related activity. For instance, if a tourist *t* walks away from his/her group to visit another POI in the surroundings then notifying the new location of *t* to the other members of his/her group can have as consequence that they will quit their current activities and move to the place where *t* is.

## 3. Functional Architecture for CAMCOS

**Figure 4.**
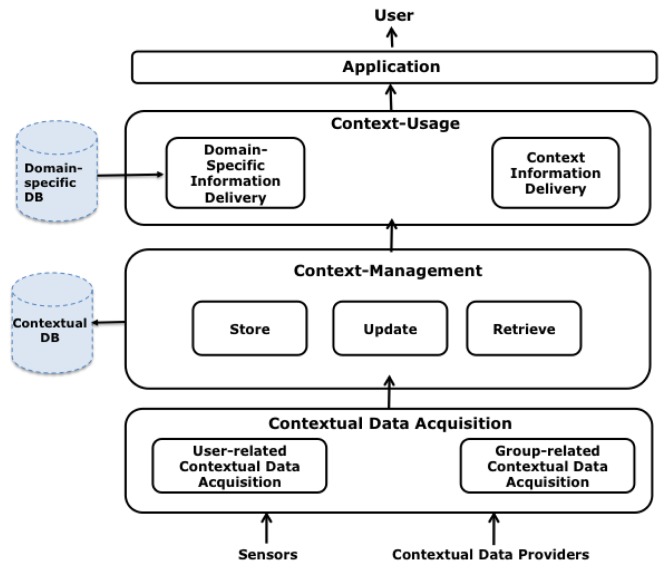
Functional architecture.

This Section presents a functional architecture for CAMCOS based on the model described (see Figure 4). It considers the main functions that any system must implement in order to be context-aware: (1) acquisition; (2) organization and management; and (3) usage of contextual data.

To acquire the current context to be used in a CAMCOS, it is necessary to obtain the contexts of the user space and of the group spaces in which the user is participating. Contextual information is retrieved from Contextual Data Sources (CDSs), each of them providing information about a logical or a physical variables. In the case of CDSs for logical variables, they take the form of providers or services that inform about the state of the variables, querying virtual or physical data repositories. In the case of CDSs for physical variables, they take the form of sensors such as a GPS receiver, a digital compass, an accelerometer, among others.

Contextual data retrieved from CDSs need to be homogeneously represented in order to ease their access and usage. First Order Logic can be used for this purpose, but other representations (relational, XML) are also possible [4]. Independently of the representation, contextual data needs to be organized in a data store from which they can be updated and retrieved. This data store can be virtual; *i.e.*, it physically does not exist but provides a common entry point to data from CDSs, or physical, with the data stored in secondary storage media. A physical data store is necessary if contextual data are to be analysed searching for behavioural patterns.

Contextual data can be basically used to provide domain-specific information to the user, but they can also be reported to the user. Consider the case of a tourist guide system: in the former case, the system can provide a user with information about a particular POI in his/her surroundings, for instance, a cathedral; in the latter case, the system can inform the user about specific interactions being executed by other users of the system, e.g., reporting that a member of a group of the user is currently visiting a specific place inside the cathedral.

## 4. The Bon Voyage Prototype System

This Section presents Bon Voyage (BV for short). First, its architecture is presented, and then its implementation and tests are explained.

### 4.1. Architecture

**Figure 5.**
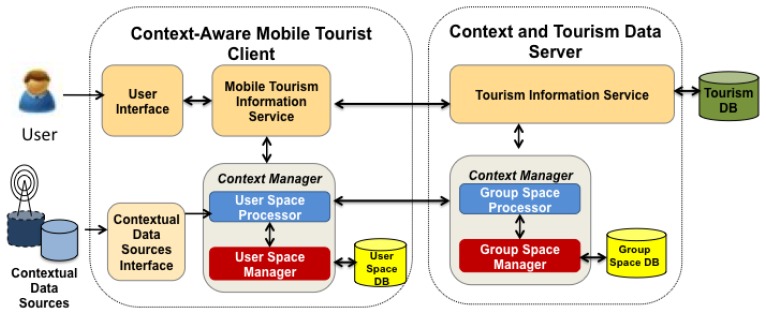
Architecture of BV.

Figure 5 shows the architecture of BV. It is basically a distributed system with a Client/Server architecture composed by a Context and Tourism Data Server, and a Context-Aware Mobile Tourist Client. The Context and Tourism Data Server has two main components: the first one is the Tourism Information Service (TIS), which manages a Tourism Database, and the second one is the Context Manager, which is distributed between the server and the mobile client. At the server side, it has two subcomponents: the Group Space Manager and the Group Space Processor. The Group Space Manager provides services to manage (create, delete, modify, query) a Group Space Database containing the elements of Group Spaces (actors, activities, objects), while the Group Space Processor interprets the rules defined in the scripts associated to the activities.

The Context-Aware Mobile Tourist Client is composed by: (i) User Interface; (ii) Contextual Data Sources Interface (CDSI); (iii) Mobile Tourism Information Service (MTIS); and (iv) Context Manager. The User Interface is the place where the input/output activity between users and the mobile device occurs, including text entry and note/map display. The CDSI component provides services to acquire and update information from contextual data sources, such as Internet services or mobile device sensors (e.g., GPS, accelerometer). The MTIS includes tools and functions, such as retrieving POIs from the Server and sharing notes, to support the tourism activities of users.

The Context Manager at the client side has two subcomponents: the User Space Manager (USM) and the User Space Processor (USP). The USM performs operations to manage (define, modify) a database containing the elements of the User Space. The USP recognizes the current user context, particularly the location of the user and his/her logical or physical activity. Logical activities include sharing a note or viewing a map, while physical activities of interest are “walking”, “standing”, “sitting”, “pointing the mobile device”. The USP retrieves basic contextual data directly from contextual data sources via the CDSI (e.g., location data is retrieved from the GPS, movement data is collected from the accelerometer) and then uses these basic contextual data to infer the user's current physical activity. The USP also interacts with the Group Space Manager to share information about user and group spaces, and provides this information to the MTIS as needed.

### 4.2. Implementation

As previously said, BV has a Client/Server architecture. The components at the server side, both the TIS and the Context Manager, were implemented as web services developed in C# on top of the Microsoft .Net Framework v3.5, and deployed in the Internet Information Services 7 web server. The Tourism DB and the Group Space DB are stored in SQL Server Express 2008.

The Mobile Client was implemented in the Android 2.2 (Froyo) platform, and uses the Ksoap2 library to call the web services at the server side. The User Interface uses parts of Mixare [5] for the AR-based version of the VNV (Other systems, such as Layar [6], can be used to implement this functionality.) The CDSI uses the Location API to get GPS data and the Hardware API to get accelerometer data. The User Space DB is stored in the SQLite DBMS. The Context Manager implements a set of simple rules that classify accelerometer measures into one of the five possible activities of interest. These rules are the result of the off-line analysis of accelerometer data collected while typical users were interacting with BV, so it was possible to manually associate physical activities to data patterns. Although this approach is limited, it was enough for testing the overall functionality of BV at this point; future versions of the system will include the usage of classifiers such as decision trees and Bayesian networks for a more flexible and automatic activity recognition [7–9].

Associated to BV, an Admin Tool and a user-friendly interface to the CDSI were also implemented. The Admin Tool enables the management of activity models, so one can specify and modify (if needed) the elements of an activity model, its preconditions and its postconditions. The CDSI Interface allows one to inspect the current data provided by the contextual data sources for debugging purposes.

### 4.3. Use Case

**Figure 6.**
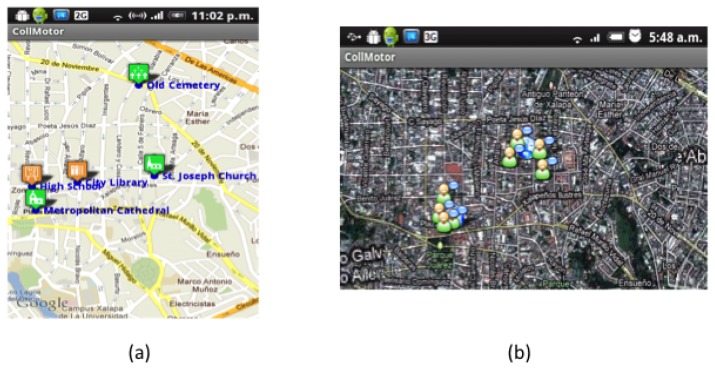
Maps showing the location of (**a**) POIs; and (**b**) tourists.

Regarding the use case, consider the previous example in which Carmen and Luis are trying to answer the question “Name of the personality who was buried in the Old Cemetery on 1938, but now is buried in the Metropolitan Cathedral”. After viewing the map indicating the location of the POIs (see Figure 6(a)), Carmen decides to go to the Cathedral while Luis visits the Old Cemetery. As Carmen goes to the Cathedral she wonders if Luis has already arrived at his destination, so she looks for him in the map displaying the current location of tourists (see Figure 6(b)) which are computed by the Context Manager at the mobile client of each user and shared with its counterpart at the server side. She sees he is almost there, so she continues her route.

**Figure 7.**
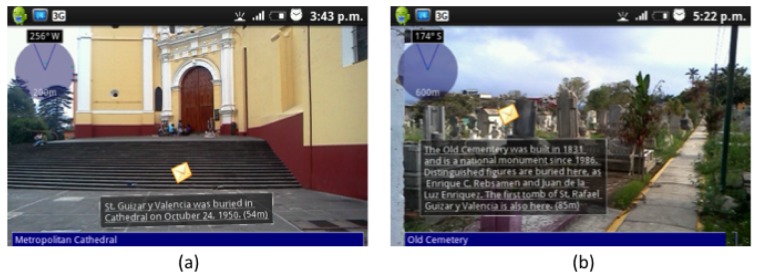
AR views associated to (**a**) “GetInfoCathedral” and (**b**) “GetInfoCemetery”.

**Figure 8.**
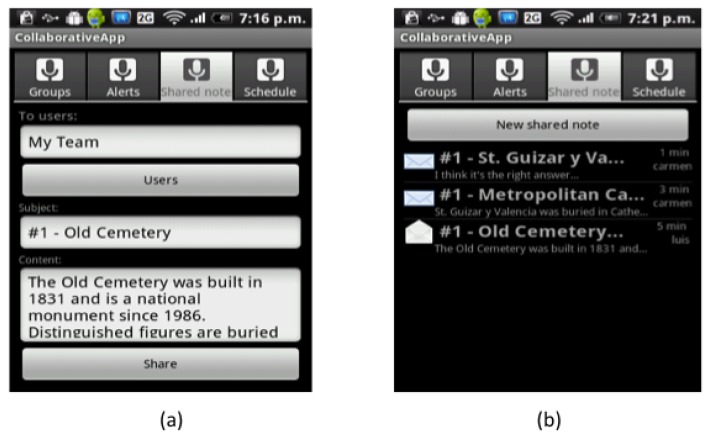
Note sharing. (**a**) Form to write a note; (**b**) Window for reading notes.

Once Carmen is in front of the Cathedral, BV detects that the “GetInfoCathedral” activity has just started in Carmen's User Space, and executes the script to present the AR-based version of the VNV (see Figure 7(a)). When Luis arrives to the Old Cemetery, something similar happens (see Figure 7(b)). He then decides to write a note to tell Carmen what he had found at the Cemetery. Figure 8(a) shows the form that Luis uses to create the note, while Figure 8(b) shows the dialog box where Carmen can see the shared note. Finally, she will answer the question with the information they found.

## 5. Evaluation

We conducted a study, which is an exploratory one, to investigate aspects related to system usability, particularly user satisfaction and system efficiency. With respect to user satisfaction, we employed subjective methods for investigating to what extent BV would be accepted by potential users (as in [10]), while concerning system efficiency, we employed objective methods to measure how the resources (particularly CPU and battery) needed for its operation are used (as in [11]). A further iteration of BV will incorporate the results of this study, after which a new evaluation is planned considering to measure system effectiveness, in terms of how well it supports its users to achieve their learning task.

The test for measuring user satisfaction was conducted with a sample consisting of 12 subjects, including students and workers. Among them, 58% were males and 42% were females, all of them between 21 and 27 years old, and with experience in the use of mobile phone devices. Before the trial begins, a general explanation of BV was given to the subjects. After that the subjects were randomly divided into 6 teams of 2 persons. The test was realized into 3 moments: at each moment, 2 teams interacted with the system. For each moment, the following steps were considered: (1) each subject was given an android-based mobile device with BV installed; (2) the teams were asked to answer correctly the questionnaire about POIs provided by the system in as little time as possible; and (3) after completing the questionnaire, each subject was asked to complete a survey with questions relating to their experience with BV. Finally, all subjects were invited to express their opinions about BV, including what worked, what did not, what features they would like to see included or any other observation.

The survey designed for this study contains 13 statements measured with a 5-point Lickert scale (see Table 3). Statements refer to four analysis dimensions: (D1) overall system satisfaction (1–3); (D2) quality of the information provided (4–7); (D3) usefulness of collaboration tools (8–11); and (D4) context-awareness (12–13). The scale used for answering the statements ranges from ‘1′, labelled ‘Strongly Disagree’, to ‘5′, labelled ‘Strongly Agree’; ‘3′ is a special case labelled ‘Neither agree nor disagree’. For example, one statement is ‘BV is easy to use’. If a subject found the system incomprehensible, he/she can score it with either a ‘1′ or a ‘2′; on the contrary, if the subject found the system really easy to use, he/she would score it with ‘5′.

**Figure 9.**
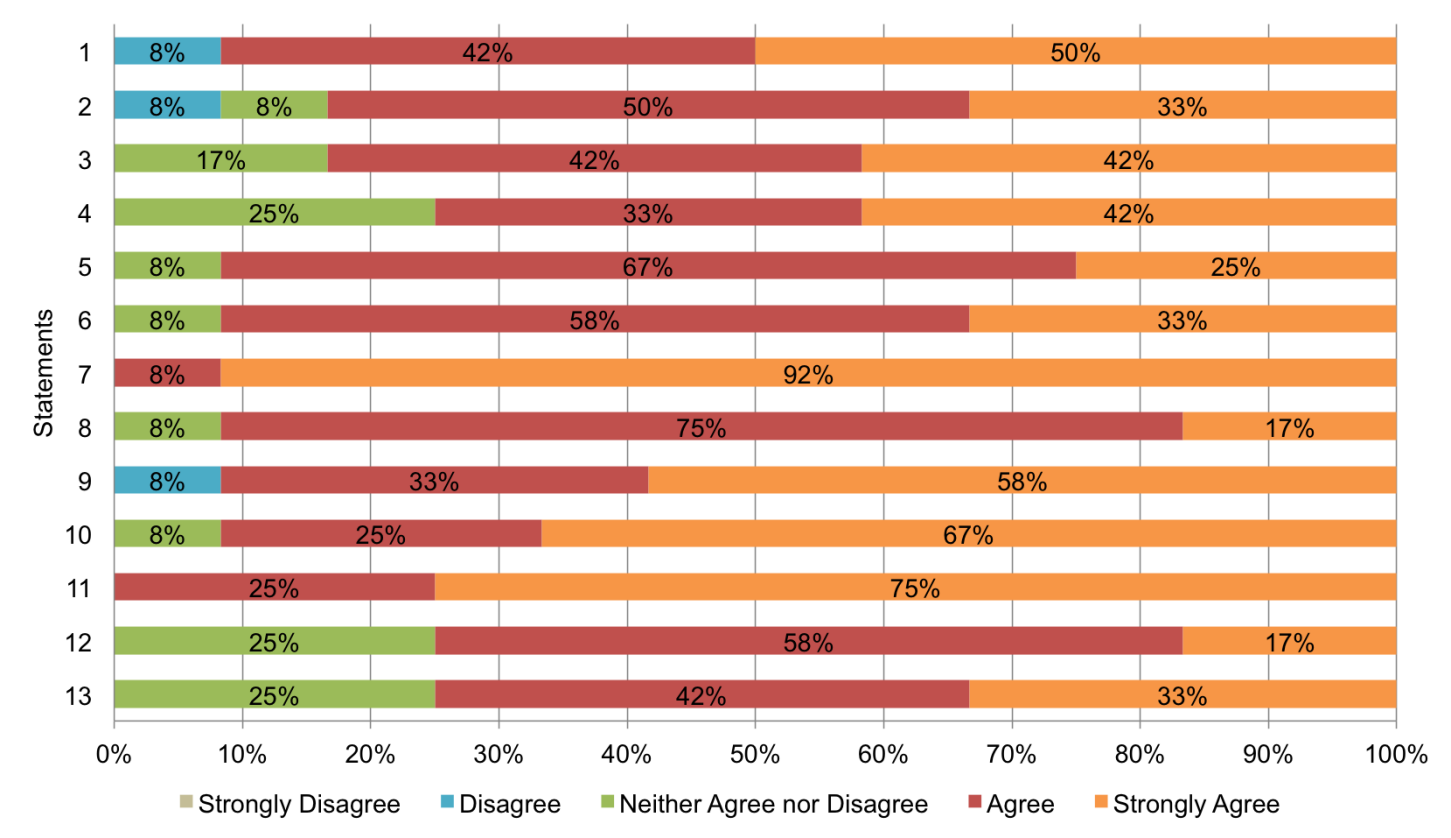
Chart summarizing the results of the survey.

Figure 9 shows a chart summarizing the results of the survey. For each statement, it indicates the opinion of the subjects regarding that statement in percentage terms; for instance, in the case of statement ‘1′ (“BV is useful to learn new things”), 8% of the participants disagree with that statement, 42% agree, and 50% strongly agree. As can be seen, in general, the subjects have well received BV: most answers are ‘agree’ or ‘strongly agree’. Regarding the analysis dimensions, it is possible to observe the following: (D1) more than 80% of the participants consider that the system satisfies his/her expectations; (D2) at least 75% of the subjects consider that the information provided in the system is of quality; (D3) more than 90% of the participants find that the collaboration tools provided by BV are useful; and (D4) at least 75% found adequate the support for context-awareness.

A detailed analysis of each item in the survey revealed the following. On the one hand, the subjects found particularly useful the maps that show the POIs and the location of users, and they were very pleased with the tool for sharing notes and with the notifications. On the other hand, 8% of the subjects (1 person) found that the system is not useful to learn new things nor it is easy to use. Besides, 25% hesitated about the context-aware features of BV. In the informal talk that we had with the participants of the trial after completing the survey, these topics were discussed. With respect to the person that stated that he had not learn anything new, he said that he expected more challenging (trivia-like) questions, as he already knew some facts about the history of the city that helped his team to answer the questions without having to visit all the POIs. Regarding the subject that said that BV is not easy use, she stated that some facilities are needed to improve the interaction with the system; for instance, she expected to easily share a virtual note attached to a POI with her team, but she was forced to write the text in a shared note. Finally, the participants that were not convinced by the context-aware features of BV indicated that they would like to have the possibility of changing the behaviour of the system to match their preferences, instead of letting the system to decide for them. The next iteration of BV will consider these suggestions to improve it.

Now, as said, one important feature of context-aware applications is self-adaptation to the current context. BV, for instance, changes the interface of the note viewer according to the physical activity of its user. But to have complete self-adapting mobile systems, other aspects related to the specific characteristics of mobile devices (particularly their limited resources) need to be considered too. Processing power and memory are becoming less important issues, but energy use is still a concern. The test for measuring system efficiency addressed this issue: its goal was to collect data about CPU load and battery consumption and analyze their behavior in order to find, if possible, patterns that could help to define adaptation rules.

**Figure 10.**
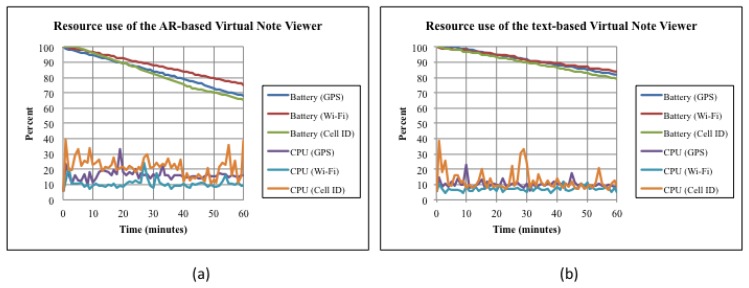
Resource use of the (**a**) AR-Based and of the (**b**) Text-based viewer.

In the experiment, data were collected while executing the Virtual Note Viewer during six one-hour periods in the same geographic area (near the Metropolitan Cathedral). During each period, one of the two versions (AR-based or text-based) of the viewer was executed using one of the three positioning technologies shown Table 4, which are available in the Android-based mobile devices used for the test. Figure 10 shows two charts that illustrate the CPU load and battery consumption of the AR-based and of the text-based VNV, respectively. From these charts, it is possible to observe the following: (i) in both cases, Wi-Fi and Cell-ID are the positioning technologies with the minimum and maximum resource use respectively; (ii) in both cases, the battery consumption of GPS and Cell-ID are similar: 32%/h and 34%/h in the AR-based version, 18%/h and 21%/h in the text-based version; (iii) in both cases, the CPU loads of GPS and Wi-Fi follows similar patterns; and (iv) as expected, the AR-based viewer uses more resources that the text-based one.

From these observations and the characteristics of positioning technologies, it is possible to conclude that: (i) GPS-based positioning uses more resources than Wi-Fi based positioning and less than Cell-ID, but it is more accurate than them; (ii) the operation requirements of the text-based version of the viewer are minimum, no matter the positioning technology used, extending battery life up to 5 hours and keeping CPU loads at low levels (ranging, in average, from 7% to 12%); however, user experience is poor; (iii) the AR viewer offers a richer user experience, but uses substantial electrical energy (a battery runs out of energy in less than 3 hours) and CPU loads reach higher levels (ranging, in average, from 10% to 21%). So, in order to offer a rich user experience and at the same time to extend battery life as much as possible, it seems reasonable to use the AR viewer with GPS-based positioning when battery levels are high, then switch to Wi-Fi based positioning when battery levels are medium (sacrificing accuracy), and finally switch to the text-based viewer with GPS-based positioning when battery levels are low (sacrificing user experience but restoring accuracy). If GPS-based positioning is not available then it is possible to switch to Wi-Fi based positioning immediately. Cell-ID should be used only when GPS and Wi-Fi signals are not available, because it is resource intensive and inaccurate.

Let us note that the application behavior described above can be captured in our model for CAMCOS as pre-conditions of the script(s) associated to an activity. For instance, the pre-conditions of the script “sCathedralAR” associated to the “GetInfoCathedral” activity can be extended to consider, besides the current physical activity of the user, the battery level. The same mechanism can be used to integrate other aspects that need to be investigated, such as use of memory, network bandwidth, and storage.

## 6. Related Work

The works related to ours can be classified in: models for CAMCOS, tools for developing context-aware systems, and context-aware tour guide systems.

Regarding models for CAMCOS, there are several proposals, some of them centered on the *activity* (e.g., [12–14]), while others are centered on the *user* (e.g., [15–19]). In [12], an activity is performed in a context, and the context is described in terms of elements with different features, such as nature, acquisition mode, relevance, and frequency of updating. [13] takes the elements of Activity Theory (AT) [20] and associate, to each of them, information about the physical environment that influences an activity; *time* is also considered to represent the evolution of the activity. [14] also proposes to associate environmental, personal, social, task and spatio-temporal information to AT elements. [15] proposes an object-oriented context representation centered on the user. This model represents both the user's physical context (location, device and application) and the user's collaborative context (group, role, member, calendar, activity, shared object and process). [16] associates a context state to users in a learning activity. A context state contains all the elements currently present within the ongoing context process that are relevant, such as the learner's current project, episode, or activity. [17] divides user context into three categories: physical, organizational and interaction. Physical context contains information about the physical situation (location, device, conditions) of users in a specific time; organizational context considers information about the user, the group, roles and tasks on specific projects; finally, interaction context includes information about synchronous or asynchronous interactions in a work group. For [18] the contextual information is categorized as static context and situation context. The static context includes human (*i.e.*, name, email, phone number) and topographic (*i.e.*, coordinates) information while the situation context includes environmental (*i.e.*, temperature, light level), personal (*i.e.*, phrase of the day), location (*i.e.*, location name) and social information. For [19] the context is composed by four dimensions: user, environment, devices and services. For each dimension, they associate information related to the 5 Ws theory (what, who, where, when and why). In this paper we focus on the description of user activities as described by the MARS model [3]. Unlike other models, ours allows the description of individual or group activities based on the notion of work rules. A work rule is described by activity models and is implemented by scripts. These scripts verify pre- and post-conditions that validate contextual aspects (personal, collaborative and awareness).

Concerning tools for developing context-aware collaborative systems, one can find middleware (e.g., [21,22]) and frameworks (e.g., [23,24]). The middleware reported in [21] is useful to get data collections from dynamically discovered hardware devices, while the one reported in [22] provides a platform based on *Smart Objects*, which can perceive its environment through sensors and communicate wirelessly with other nearby objects. Regarding frameworks, [23] proposes a framework enabling the configuration of context providers based on automatic reasoning, while [24] proposes a framework of collaboration services based on a hybrid model of context management. These proposals are important, however, they focus on enabling low-level collaboration (e.g., communication protocols) among devices. Instead, our work approaches the problem from a social perspective and is aimed to ease the collaboration among people, where communication, coordination and cooperation are needed to complete a group activity.

Finally, it is possible to find other proposals of context-aware mobile applications for tourism, for instance [25–28]. These works are mainly based on location, while our work includes also social aspects during the execution of an activity, such as collaboration and awareness. In this sense, it is closer to works such as Musex [29], which is an interactive guide system that was created to familiarize children with exhibitions at a Museum, providing children with opportunities to answer collaboratively questions related to exhibitions. BV supports a collaborative activity similar to the orienteering game of Musex, but it is not confined to a museum, being the city the play field. Besides, BV is more flexible than Musex, thanks to the ability of adapting itself.

## 7. Conclusions and Future Work

This paper proposes a novel activity model for context-aware mobile collaborative systems. It is based on the notions of user and group spaces, where users execute activities, either in isolation or within a work group respectively, under a certain context. Associated to this model, a functional architecture for this kind of systems is also proposed. In order to test our approach, the prototype system *Bon Vogage*, implementing the model and instantiating the functional architecture, was built. The exploratory study we conducted to test Bon Voyage revealed a high level of user satisfaction and the potential for resource-driven self-adaptation, but it will be necessary to conduct thorough studies including also effectiveness aspects.

Our future work includes extending the model to enable the representation of more complex scenarios, and adapting the functional architecture and the prototype accordingly to include this model extension. It also includes improving the user interface and functionalities of BV. Particularly, it is necessary (a) to include more challenging questions; (b) to integrate additional facilities to ease human-system interactions; such as enabling a user to share location-attached notes with other users with just one click; (c) to incorporate more robust physical activity detection mechanisms; and (d) to give users the possibility of modifying the scripts associated to an activity in order to match their preferences.

## Figures and Tables

**Table 1.** Elements of the model for CAMCOS.

**Element**	**Description**
Activity	An activity in execution
Actor	A person, a software agent or a group that executes (or participates on) an activity
Object	An object manipulated or produced during an activity
Family	Group of actors/objects with similar features
Role	Associated to actors and objects during an activity
Script	Description of how an activity can be executed
Activity Model	Generic description of an activity

**Table 2.** Example of activity model.

**Element**	**Description**
Name	GetInfoCathedralModel
Family	Tourist for actors Document for objects
Roles	Reader for Tourist BeingRead for Document
Script	s1: sCathedralTxt s2: sCathedralAR

**Table 3.** Relation of statements in the survey.

**No.**	**Dimension**	**Statement**
1	D1	BV is useful to learn new things
2	D1	BV is easy to use
3	D1	BV enriched my tourist activity
4	D2	The questions are interesting
5	D2	The virtual notes attached to POIs are useful
6	D2	The clues associated to the questions are useful
7	D2	The map showing the location of POIs is useful
8	D3	The Shared Notes tool is useful
9	D3	The notifications are relevant
10	D3	The map showing the location of tourists is useful
11	D3	BV offers the right tools for team collaboration
12	D4	The User Interface adapts adequately to my current physical activity
13	D4	Notifications about the activities executed by other tourists affect my activity

**Table 4.** Characteristics of positioning technologies.

**Positioning Technology**	**Accuracy**	**Reference**	**Limitation**
GPS	High (3–10 m)	Absolute	For outdoors only
Wi-Fi	Medium (10–100 m)	Relative to an access point	Access point availability
Cell-ID	Low (100 m–3 km)	Relative to a GSM tower	GSM network availability
